# Practical Manual of Obstetrics

**Published:** 1884-09

**Authors:** 


Practical Manual of Obstetrics. Dr. E. Verrier, Lecturer on Obstetrics in the Faculty of Medicine of Paris. Fourth edition, enlarged and revised. First American edition with revision and annotations by Edward L. Partridge, M.D., Professor of Obstetrics in the New York Post-graduate Medical School. William Wood & Co., New York, 1884.

It gives one a favorable impression of a French obstetrician that he should not think all law and gospel that was inculcated by the extensive and yet comparatively unprofitable experience of Madame Le Chapelle, yet the subserviency to old usage is manifested by our author in gravely detailing the order in which the three different caps are put upon the head of a new-born infant.

In recommending fifteen drops of laudanum with injections of warm water every fifteen minutes in hemorrhage connected with threatened abortion, it is forgotten that small doses of opium prove stimulant, while if a dose of fifty drops of laudanum by the mouth is given every hour or two a sedative effect is secured.

In face presentation, with the vertex to the front, our author very glibly states that "when rotation does not occur, rotation of the chin forward may be produced by a couple of applications of the forceps," but many a practical obstetrician has failed, nd others will fail in this maneuvre.

The only effectual proceeding is to bring about entire relaxation of the womb by chloroform, return the head into the womb and correct it, so as to bring down the vertex, or by podalic version.

The best suggestion contained in this volume is in regard to the management of the head to obviate rupture of the perineum, by the American editor, and while saying some absurd things he enjoins the attendant to "be careful also to disengage the occiput from the pubic arch and anterior vulval commission, so that that portion of the head shall be wholly delivered before allowing descent and extension of the face." The true rationale of this procedure is not given, and consists in bringing the head into a conoidal form, which may be formed by the grasping, so far as may be practicable, around the most projecting part outside of the perineum. Every obstetrician will have noted the lengthening of the head in vertex presentations accompanied with great resistance of the soft parts from the lapping of the parietal bones, and so far as this result can be secured by compression with the hands over the distended perineum, there will be less risk of laceration.

The author's treatment of retained placenta is so ill-defined that it may be pardonable to submit the recourse of prompt and complete extraction by the hand in all cases not spontaneously delivered or expelled by the Credè method within half an hour after the birth of the child.

Notwithstanding its faults, the book may be read with profit.

